# HDL nanoparticles targeting sonic hedgehog subtype medulloblastoma

**DOI:** 10.1038/s41598-017-18100-8

**Published:** 2018-01-19

**Authors:** Jonathan B. Bell, Jonathan S. Rink, Frank Eckerdt, Jessica Clymer, Stewart Goldman, C. Shad Thaxton, Leonidas C. Platanias

**Affiliations:** 1 Robert H. Lurie Comprehensive Cancer Center of Northwestern University, Lurie 3-125, 303 E. Superior St., Chicago, IL 60611 United States; 2 Department of Urology, Feinberg School of Medicine, Northwestern University, 303 E. Superior St., Chicago, IL 60611 United States; 3 Simpson Querrey Institute (SQI) for BioNanotechnology, 303 E. Superior St., Chicago, IL 60611 United States; 4 Department of Neurological Surgery, Feinberg School of Medicine, Northwestern University, 303 E. Superior St., Chicago, IL 60611 United States; 5 Division of Hematology/Oncology/Stem Cell Transplantation, Department of Pediatrics, Ann & Robert H. Lurie Children’s Hospital of Chicago, 225 E. Chicago Ave., Chicago, IL 60611 United States; 6 Division of Hematology/Oncology, Department of Medicine, Feinberg School of Medicine, 303 E. Superior St., Chicago, IL 60611 United States; 7 Department of Medicine, Jesse Brown VA Medical Center, 820S. Damen Ave., Chicago, IL 60612 United States

## Abstract

Medulloblastoma is the most common paediatric malignant brain cancer and there is a need for new targeted therapeutic approaches to more effectively treat these malignant tumours, which can be divided into four molecular subtypes. Here, we focus on targeting sonic hedgehog (SHH) subtype medulloblastoma, which accounts for approximately 25% of all cases. The SHH subtype relies upon cholesterol signalling for tumour growth and maintenance of tumour-initiating cancer stem cells (CSCs). To target cholesterol signalling, we employed biomimetic high-density lipoprotein nanoparticles (HDL NPs) which bind to the HDL receptor, scavenger receptor type B-1 (SCARB1), depriving cells of natural HDL and their cholesterol cargo. We demonstrate uptake of HDL NPs in SCARB1 expressing medulloblastoma cells and depletion of cholesterol levels in cancer cells. HDL NPs potently blocked proliferation of medulloblastoma cells, as well as hedgehog-driven Ewing sarcoma cells. Furthermore, HDL NPs disrupted colony formation in medulloblastoma and depleted CSC populations in medulloblastoma and Ewing sarcoma. Altogether, our findings provide proof of principle for the development of a novel targeted approach for the treatment of medulloblastoma using HDL NPs. These findings present HDL-mimetic nanoparticles as a promising therapy for sonic hedgehog (SHH) subtype medulloblastoma and possibly other hedgehog-driven cancers.

## Introduction

Medulloblastoma is the most common malignant brain tumour in children, with a particularly poor prognosis for patients with relapsing disease^1,2^. Although young children experience poor outcomes, treatment options are often limited due to the concern for the development of neurocognitive deficits following chemotherapy and radiation^3^. Undoubtedly, new therapies are needed, particularly for younger children and children with recurrent disease. In addition to histopathological classifications, medulloblastoma has been subdivided into four molecular subtypes in the updated WHO classification system for CNS tumours^4^. These subtypes include group 3, group 4, wingless (WNT) and sonic hedgehog (SHH) medulloblastoma^5–7^, each of which relies upon different molecular drivers and signalling pathways, allowing for targeting specific molecular subtypes. SHH subtype medulloblastoma is driven by binding of the hedgehog ligands Sonic Hedgehog (SHH), Indian Hedgehog (IHH), or Desert Hedgehog (DHH) to the Patched (PTCH) transmembrane receptor^8^. In turn, this leads to dis-inhibition of Smoothened (SMO), GLI translocation to the nucleus and transcription of GLI-target genes^9^. Cholesterol plays a central role in the regulation of SHH signalling in medulloblastoma and normal cells. Hedgehog ligands and SMO are the only known proteins to undergo covalent modification by cholesterol (i.e., cholesterylation)^10,11^. Furthermore, cholesterol directly binds the extracellular, cysteine-rich domain (CRD) of SMO, leading to its activation^12^. Therefore, disruption of cholesterol signalling may be an effective strategy in hedgehog-driven cancers, including medulloblastoma.

Bio-inspired, synthetic HDL nanoparticles (HDL NPs) are similar in size, shape, charge, surface composition and share some similar functions to natural HDLs and have been shown to inhibit the growth of lymphoma and leukaemia through disruption of normal cholesterol homeostasis^13–15^. HDL NPs bind to the high-affinity HDL receptor scavenger receptor type B1, SCARB1, promoting cholesterol efflux and disrupting cellular cholesterol levels, leading to subsequent lymphoma cell death.

Here, we examine the effect of HDL NPs on the growth of SHH-subtype medulloblastoma. Analysis of a large cohort of medulloblastoma patients indicates that SCARB1 is expressed in medulloblastoma cells and particularly enriched in the SHH subtype. Furthermore, we demonstrate binding of the HDL NPs to cells in medulloblastoma, as well as Ewing sarcoma, another hedgehog-driven cancer. Using fluorescently labelled HDL NPs, we show internalization of the nanoparticles in medulloblastoma cells. Finally, we show depletion of cell viability, colony formation and cancer stem cell frequencies in medulloblastoma and Ewing sarcoma in response to treatment with these nanoparticles. Together, these findings suggest a promising role for HDL NPs in the treatment of medulloblastoma and other hedgehog-driven cancers.

## Results

### The role of SCARB1 in hedgehog-subtype medulloblastoma

**Figure 1:**
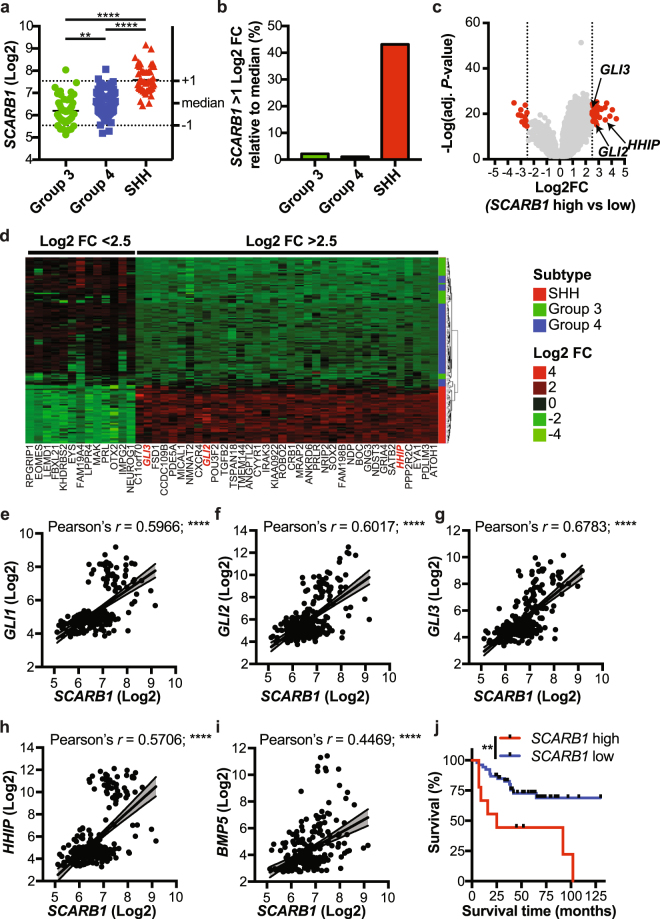
HDL receptor, *SCARB1*, is overexpressed in sonic hedgehog driven medulloblastoma and high expression predicts poor survival. (**a**) *SCARB1* expression was analysed in different medulloblastoma subtypes from the Northcott dataset^30^, one-way ANOVA, **P ≤ 0.01, ****P ≤ 0.0001. (**b**) Percent of patients in different subtypes from the Northcott dataset with *SCARB1* > 1 Log2 FC relative to the median expression. (**c**) Differential expression analysis of medulloblastoma patient samples with high vs. low *SCARB1* expression from the Northcott dataset. (**d**) Heatmap showing differential expression in medulloblastoma patients as in (**c**). (**e**–**i**) Gene expression data was analysed by correlation analysis. *SCARB1* expression is compared with five SHH pathway genes *GLI1* (**e**), *GLI2* (f), *GLI3* (**g**), *HHIP* (**h**) and *BMP5* (**i**). Pearson’s correlation coefficients are shown, ****P ≤ 0.0001. (**j**) Survival data from the Pomeroy dataset^31^ was analysed by Kaplan Meier curve comparison (n = 62). Log-rank (Mantel-Cox), *P* = 0.0039.

The role of cholesterol in the regulation of sonic hedgehog (SHH) signalling has been previously well described^11,16^. However, the expression of the HDL-receptor, SCARB1, in SHH-driven cancers has not been studied. We analysed the expression of the *SCARB1* gene in medulloblastoma, a tumour driven by aberrant activation of the SHH signalling pathway. Analysis using the Northcott dataset revealed that *SCARB1* was most highly expressed in SHH subtype medulloblastoma (Fig. 1a) with a significant number of SHH subtype patient samples expressing *SCARB1* >1 Log2 fold change when compared to the median (i.e., greater than twice the median expression value) (Fig. 1b). Furthermore, analysis of patient samples revealed an enrichment of several hedgehog pathway genes, such as *GLI2*, *GLI3* and *HHIP* in cells from medulloblastoma patients expressing high *SCARB1* levels (Fig. 1c,d). *SCARB1* expression was also found to correlate positively with expression of several hedgehog genes (Fig. 1e–i). Conversely, SCARB1 expression negatively correlated with several non-SHH genes found to be underexpressed in *SCARB1* low patient samples (Fig. S1). As *SCARB1* is a negative prognostic factor in several cancers^17,18^, we next analysed the effect of *SCARB1* on patient outcomes in medulloblastoma. High expression of *SCARB1* was associated with shorter overall survival in medulloblastoma patients (Fig. 1j). These findings suggest a significant role for the HDL signalling and SCARB1 receptor in SHH subtype medulloblastoma.

### Binding and uptake of HDL NPs

**Figure 2:**
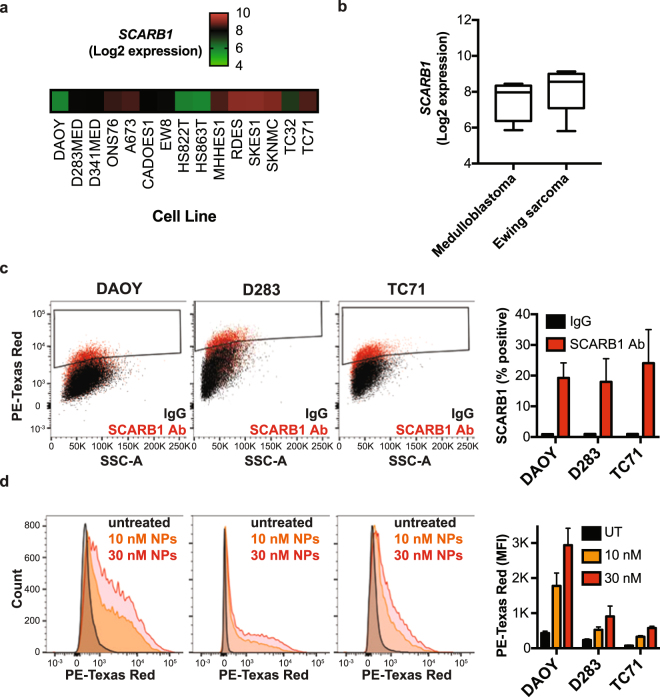
HDL NPs bind to SCARB1-expressing medulloblastoma and Ewing sarcoma cells. (**a,b**) *SCARB1* expression from a panel of medulloblastoma and Ewing sarcoma cell lines was downloaded from the Cancer Cell Line Encyclopedia (CCLE). **(c**) DAOY, D283 and TC71 cells were stained with the SCARB1 antibody or IgG isotype control and analysed by flow cytometry. Representative dot plots and a bar graph depicting the percentage of SCARB1 positive cells are shown. Data represent means ± SEM of 3 independent experiments. (**d**) DAOY, D283 and TC71 cells were treated with increasing concentrations of DiI-labelled HDL NPs for two hours and analysed by flow cytometry. Representative histograms and a bar graph depicting the median fluorescence intensity (MFI) are shown. Data represent means ± SEM of 2–4 independent experiments.

**Figure 3:**
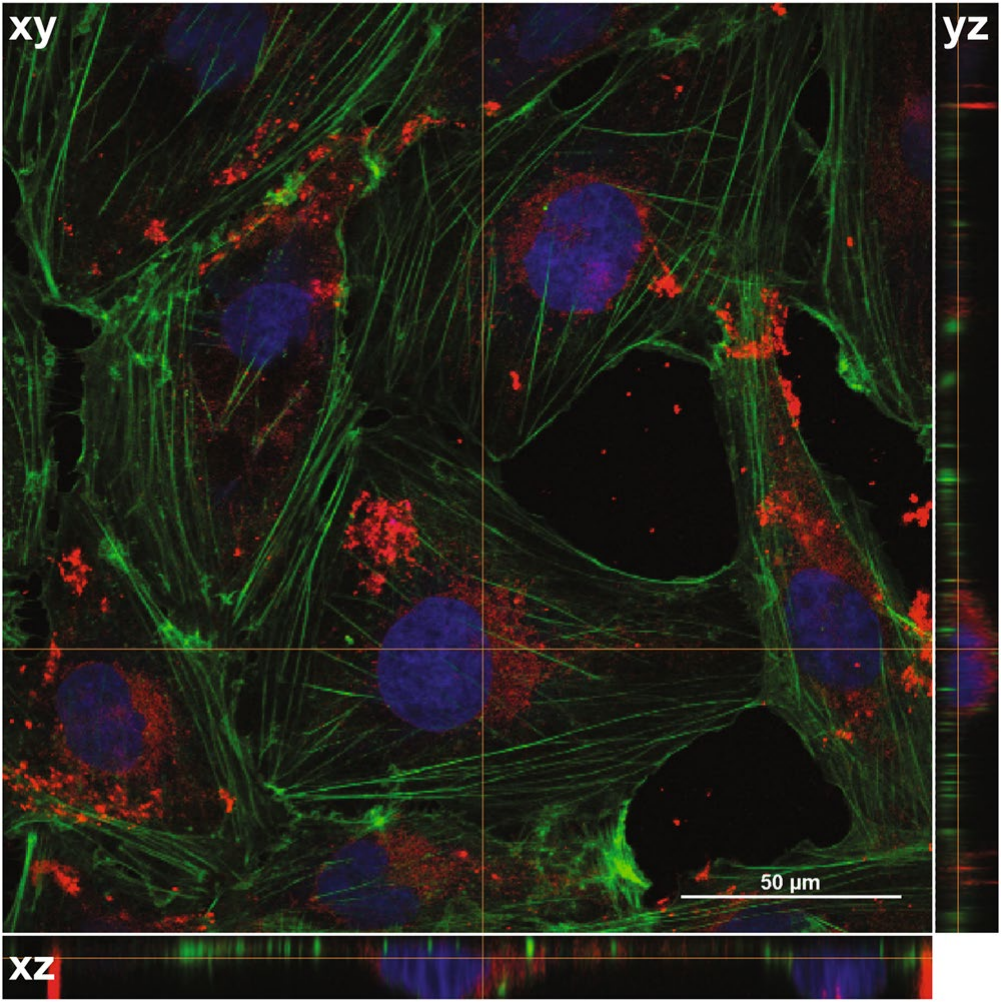
Uptake of HDL NPs in medulloblastoma cells. Confocal images of DAOY cells treated with 30 nM DiI labeled HD NPs for 24 hours. Images were composed from 0.18 μm serial confocal images (16 slices) through the z-plane of the cells. Representative orthogonal-slice views show cell nuclei (blue), actin (green) and HDL NPs (red). The middle, right and bottom panels are the xy, yz and xz planes, respectively. The yz and xz planes intersect according to the crosshairs. Scale bar = 50 μm.

After uncovering a potential role for the HDL receptor, SCARB1, in SHH-driven medulloblastoma, we tested the effect of synthetic HDL NPs on SHH-driven cancer cells. First, we analysed *SCARB1* expression in a panel of medulloblastoma and Ewing sarcoma cell lines from the Cancer Cell Line Encyclopedia (Fig. 2a,b)^19^. These cell lines expressed *SCARB1*, supporting our findings from medulloblastoma patient samples. In addition, flow cytometry analysis revealed the presence of this receptor on the surface of our medulloblastoma cell lines as well as a SHH-driven Ewing sarcoma cell line (Fig. 2c). Next, we sought to analyse binding of HDL NPs to SCARB1 expressing cells. Using fluorescently-labelled HDL NPs and flow cytometry, we found an increase in fluorescence intensity upon HDL NP treatment, suggesting binding of the particles to these cells (Fig. 2d). We next sought to investigate uptake of the HDL NPs in our cells. Using confocal laserscan microscopy we found fluorescently-labelled nanoparticles accumulated in medulloblastoma cells (Fig. 3). This accumulation was dose-dependent with highest concentrations in the perinuclear area, suggesting efficient uptake and internalization of the HDL NPs in cells (Figs 3 and S2). These data confirm expression of the SCARB1 protein in our medulloblastoma and Ewing sarcoma cells and demonstrate effective binding and uptake of the HDL NPs.

### Antitumour effects of HDL NPs in medulloblastoma

**Figure 4:**
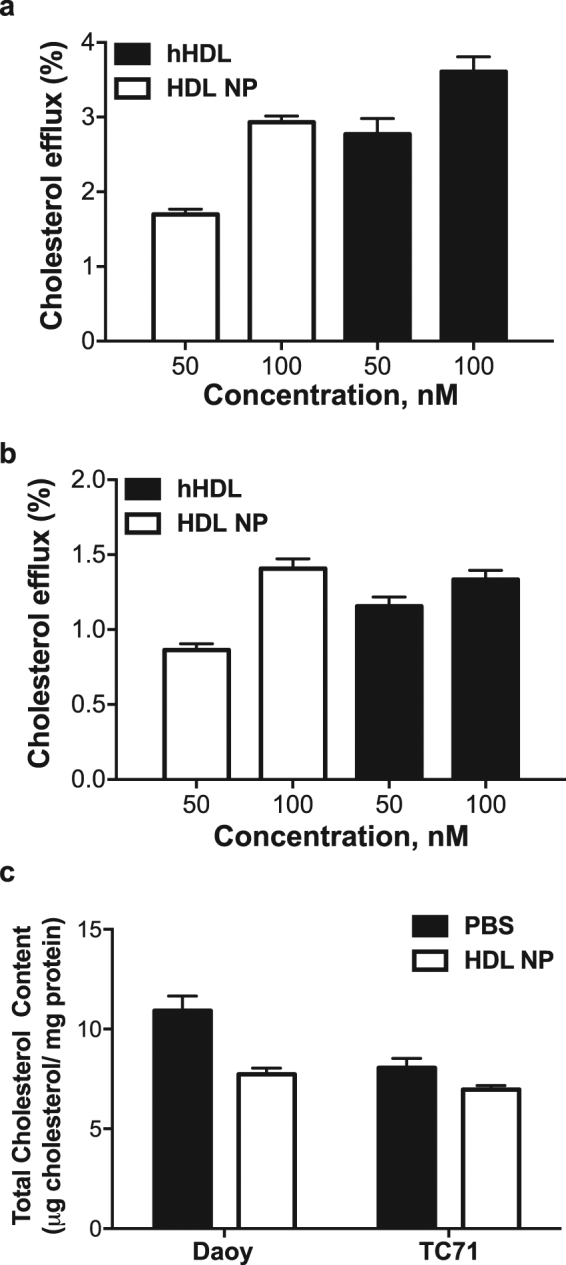
HDL NPs differentially modulate cellular cholesterol in medulloblastoma and Ewing sarcoma cells. (**a**,**b**) DAOY (**a**) or TC71 (**b**) cells were incubated with [1,2–^3^H] cholesterol overnight followed by incubation with normal human HDL (hHDL) or HDL NPs at different concentrations for 4 hours. Cholesterol efflux was determined by liquid scintillation counting. Data presented as means ± SD of 3 biological replicates. (**c**) Total cholesterol levels in DAOY and TC71 cells following 24-hour treatment with HDL NPs was determined using the Amplex Red Cholesterol Assay. Data are standardized to mg protein in each sample and are presented as means ± SD of 3 biological replicates.

**Figure 5:**
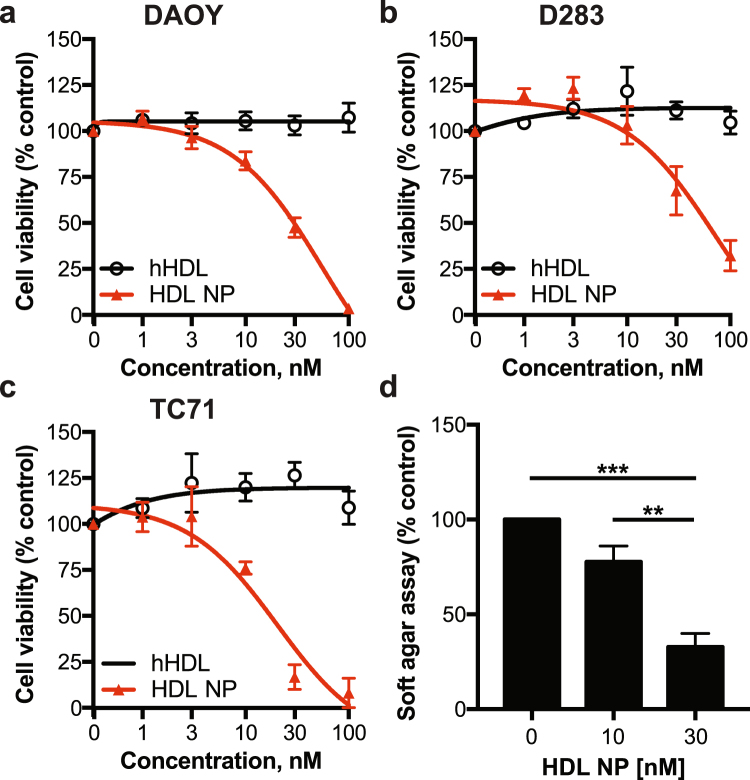
HDL NPs inhibit viability and colony formation. (**a,c**) DAOY, D283 and TC71 cells were treated with increasing concentrations of HDL NPs or natural human HDL (hHDL) for 5 days followed by analysis with the WST-1 cell proliferation assay. Data represent means ± SEM of 2–4 independent experiments. **(d)** DAOY cells were seeded in soft agar and treated with HDL NPs at the indicated concentrations for 7 days followed by staining with the fluorescent CyQUANT GR Dye. Data represent means ± SEM of 3 independent experiments. One-way ANOVA, **P ≤ 0.01, ***P ≤ 0.001.

**Figure 6:**
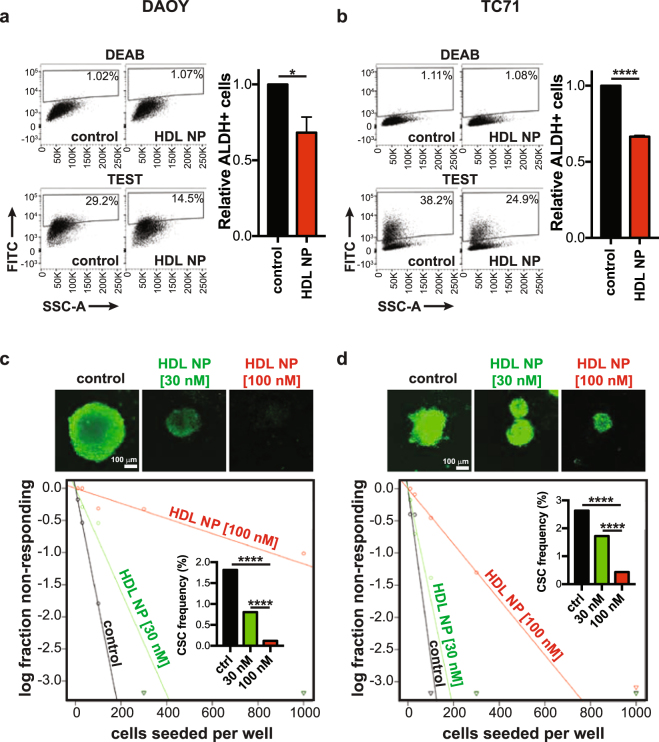
Depletion of cancer stem cell populations with HDL NPs. (**a,b)** DAOY and TC71 cells were treated with or without HDL NPs (100 nM) for 2 days followed by analysis with the ALDEFLUOR Kit. Cells were stained with the ALDEFLUOR reagent in the presence (top) or absence (bottom) of the ALDH inhibitor, DEAB, to determine the frequency of ALDH+ cells. Representative dot plots (left panels) and bar graphs (right panels) depicting the proportion of ALDH+ cells relative to control are shown. Data represent means ± SEM of 3 or 4 independent experiments. Unpaired, two-tailed t-test, *P ≤ 0.05, ****P ≤ 0.0001. (**c,d)** DAOY or TC71 cells were grown as spheres in CSC medium for one week. Spheres were then dissociated and seeded at increasing cell numbers into round-bottom 96-well plates in the presence or absence of HDL NPs to assess sphere formation. After one week, spheres were stained with acridine orange and imaged. Spheres ≥100 μm were counted and analysed by extreme limiting dilution analysis (ELDA) to determine CSC frequency, χ^2^: ****P ≤ 0.0001. Data from 4 independent experiments.

We next tested the effect of HDL NPs on cholesterol efflux and total cholesterol in SHH driven medulloblastoma and Ewing sarcoma cells. After treatment of cells with HDL NPs we observed a dose-dependent increase in cholesterol efflux, similar to the trend observed upon human HDL (hHDL) treatment (Fig. 4a,b). We also found that treatment of both cell lines led to a decrease in total cholesterol (Fig. 4c), in line with our findings from other cancers^13^. Given the effect of the HDL NPs on cholesterol efflux and total cholesterol, we next assessed their effect on viability and colony formation in soft agar. HDL NPs disrupted cell viability in two medulloblastoma cell lines and one Ewing sarcoma cell line, in a dose-dependent manner (Fig. 5a–c). Used at the same concentrations, hHDL did not affect cell viability in these cells, indicating that the effects of the nanoparticles on cell viability are specific to the nanoparticles. Finally, HDL NPs potently disrupted colony formation in soft agar in SHH-driven medulloblastoma cells (Fig. 5d). Notably, the anti-proliferative effect of HDL NPs was not seen in D556 cells, a medulloblastoma cell line driven by *MYCC* amplification (Fig. S3)^20,21^. Persistence of cancer stem cells (CSC) is believed to be responsible for tumour recurrence after standard chemotherapy and radiation in medulloblastoma and other brain tumours. Targeting this population is likely important to achieve lasting tumour remission. Therefore, we next tested the effect of HDL NPs on the CSC frequency in medulloblastoma and Ewing sarcoma. After treatment with the HDL NPs, we found a marked decrease in the number of ALDEFLUOR positive (ALDH+) cells (Fig. 6a,b). We also found that treatment with HDL NPs depleted the CSC frequency of these cell lines when the cells were grown, as 3-D spheres, in CSC medium (Fig. 6c,d).

## Discussion

HDL NPs are a promising nanotherapeutic approach with potential implications for several cancer types. Tumours that rely upon cholesterol and HDL signalling and which express the HDL receptor SCARB1, may be particularly sensitive to these nanoparticles^13,22^. Expression of SCARB1 drives tumour growth in a number of different cancers including breast and prostate cancer^23,24^. Targeting SCARB1 expressing cells with HDL NPs represents a promising strategy to treat a number of different cancer types that rely upon HDL signalling.

SHH-subtype medulloblastoma relies upon HDL to promote bulk tumour growth as well as maintain tumour-initiating CSC populations^9^. In line with this we found that overexpression of SCARB1 in medulloblastoma corresponds to poor prognosis. We demonstrate that SHH-subtype medulloblastoma overexpresses SCARB1 when compared with other molecular subtypes. Flow cytometry analysis indicates the receptor is present on the cell surface and likely facilitates binding to HDL NPs. HDL NPs engage the SCARB1 receptor similar to hHDL as demonstrated by increased cholesterol efflux, resulting in cellular internalization of nanoparticles. Consequentially, biomimetic HDL NPs, exhibited potent antineoplastic effects against medulloblastoma and hedgehog-driven tumour Ewing sarcoma cells. Importantly, HDL NPs reduce the number of ALDH+ cells and disrupt stem cell frequencies in the SHH-driven medulloblastoma and Ewing sarcoma cell lines indicative of potent inhibitory effects on the CSC populations in these cancers. These data suggest that HDL NPs may specifically deplete these tumour-initiating cells, thus preventing tumour recurrence and therapeutic resistance.

Medulloblastoma is a tumour that arises exclusively in the cerebellum, a critical region of the brain located in the posterior cranial fossa^25^. As with other brain tumours, the blood-brain barrier (BBB), a physical and biochemical barrier that prevents the delivery of therapeutics to the central nervous system, protects the tumour^26^. Recently, it has been shown that different medulloblastoma molecular subtypes have more or less intact BBBs, a factor that will need to be considered when designing nanotherapeutics for medulloblastoma^26^. Importantly, *in vivo* studies with tumour-bearing mice will be needed to provide insight into whether HDL NPs can reach medulloblastoma tumours at an effective dose. In addition to the intrinsic antitumour properties of HDL NPs, there is the potential for the development of novel nanoconjugates using HDL NPs as a scaffold for the delivery of oncogene-targeting small interfering RNAs (siRNAs)^27,28^. In medulloblastoma, HDL NPs conjugates could be designed to deliver therapies that complement the effect of the particles, potentially synergizing with the effect of the HDL NPs. For SHH driven medulloblastoma, potential targets include the GLI transcription factors, proteins difficult to inhibit with small molecules^9^.

In summary, we present a novel application of HDL NPs for the treatment of SHH-driven medulloblastoma. We demonstrate binding and uptake of these nanoparticles in cells that express the HDL receptor, SCARB1. We also show anti-neoplastic effects in medulloblastoma as well as a second hedgehog-driven cancer type, Ewing sarcoma. These findings provide evidence for targeting hedgehog-driven cancers using these novel biomimetic nanoparticles and further evidence in that direction are warranted.

## Methods

### Bioinformatics

Gene expression data from medulloblastoma patients was analysed using the GlioVis software^29^. To analyse SCARB1 expression in medulloblastoma subtypes and identify genes overexpressed in *SCARB1* high expressing medulloblastoma patients, the “Northcott_2012” dataset was used with a 2.5X Log2 fold change cut-off for differentially expressed genes^30^. Correlation of *SCARB1* with hedgehog and non-hedgehog pathway genes was performed using Pearson correlation analysis. The “Pomeroy” dataset^31^ was used for survival analysis in *SCARB1* high and low-expressing patients. *SCARB1* expression in medulloblastoma and Ewing sarcoma cell lines was downloaded from the Cancer Cell Line Encyclopedia (CCLE)^19^.

### Cell culture

DAOY and D283 cells were grown in DMEM supplemented with 10% FBS (Thermo Fisher) and gentamycin (1 mg/mL). TC71 cells were grown in RPMI supplemented with 10% FBS and gentamycin (1 mg/mL). Cell lines were authenticated in March 2017 by short-tandem repeat (STR) analysis (Genetica DNA Laboratories). To establish 3-D cancer stem cell (CSC) cultures, 5 × 10^6^ DAOY or TC71 cells were seeded in non-adherent T75 flasks containing CSC medium consisting of DMEM/F12 supplemented with EGF (20 ng/mL), bFGF (20 ng/mL), heparin (5 μg/mL), B27 (2%) and gentamycin (1 mg/mL). Cells were propagated in CSC medium for one week prior to sphere forming assay.

### Synthesis of HDL NPs

HDL NPs were synthesized as previously described^13,32–34^. Briefly, 5 nm citrate-stabilized gold colloid was surface functionalized by addition of apolipoprotein A-I (ApoA-I; 5-fold molar excess relative to gold colloid) and the phospholipids PDP PE (1,2-dipalmitoyl-*sn*-glycero-3-phosphoethanolamine-N-[3-(2-pyridyldithio)propionate]; 250-fold molar excess) and DPPC (1,2-dipalmiotyl-*sn*-glycero-3-phosphocholine; 250-fold molar excess). HDL NPs were purified by tangential flow filtration and the concentration determined by UV/Vis spectroscopy and Beer’s law. For the fluorescently-labelled HDL NPs, the intercalating dye (1,1′-Dioctadecyl-3,3,3′,3′-Tetramethylindocarbocyanine Perchlorate; 12.5-fold molar excess) was added to the synthesis during phospholipid addition.

### Flow cytometry

To assess SCARB1 expression, cells were incubated with PE anti-human CD36L1 (SCARB1, SR-BI) Antibody or mouse IgG1, κ isotype control (BioLegend) for one hour at room temperature. Following antibody incubation, cells were washed three times with PBS and analysed by flow cytometry. To assess nanoparticle binding, cells were treated with 1,1′-Dioctadecyl-3,3,3′,3′-Tetramethylindocarbocyanine Perchlorate (DiI)-labelled HDL NPs at the indicated doses for three hours at 37 °C. Cells were then washed three times with PBS and analysed by flow cytometry. To assess ALDH activity, cells were analysed using the ALDEFLUOR Assay (Stem Cell Technologies) according to the manufacturer’s instructions. Cells were treated with HDL NPs or vehicle control (PBS) for three days. Cells were then washed with PBS, re-suspended in Assay Buffer, stained with the ALDEFLUOR reagent and half of each sample was immediately transferred to a tube containing the ALDH inhibitor, Diethylaminobenzaldehyde (DEAB). Cells were then incubated for 30 minutes at 37 °C. Subsequently, cells were washed three times with PBS and analysed by flow cytometry. All flow cytometry analysis was performed using FlowJo 10 for Mac.

### Confocal laserscan microscopy

DAOY cells were incubated with DiI-labelled HDL NPs (red) for 24 hours at 37 °C. For live cell imaging, cells were seeded onto glass-bottom dishes (MatTek). For all other microscopic analyses, cells were seeded onto 0.17 mm coverslips (Electron Microscopy Sciences), fixed with 4% paraformaldehyde (PFA) and incubated with DAPI to visualize nuclei (blue) or phalloidin-AF488 to visualize actin (green). Microscopy was performed with a Nikon A1R+ inverted microscope with an Apo 60X oil objective lens from Nikon with NA 1.4. For microscopic analysis, the acquisition software NIS-Elements was used.

### Cholesterol efflux assay

The cholesterol efflux assay was performed as previously reported^35^. Daoy and TC71 cells were plated into 24 well plates at a density of 1.5 × 10^5^ cells per well. Cells were radiolabelled with ^3^H-cholesterol (1 μCi/ml culture media; PerkinElmer) for 24 hours. Cells were then washed twice with serum-free culture media and the cholesterol effluxors added in serum-free media for 4 hours. HDL NPs were added at a concentration of 50 or 100 nM. Human HDL was added at an equivalent concentration based on apolipoprotein A-I content. After incubation, cells were spun at 1000 rpm for 4 minutes and the supernatants filtered prior to liquid scintillation counting. To quantify the total radiolabelled cholesterol, cells were spun down prior to cholesterol acceptor addition and resuspended in isopropanol. The resultant isopropanol solution was then dried under nitrogen gas and the ^3^H-cholesterol dissolved into xylene, prior to liquid scintillation counting. The percentage of cholesterol efflux was determined for each well using the formula: counts_(supernatant)_/counts_(total)_ × 100. The background cholesterol efflux obtained in the absence of any acceptor (PBS control) was subtracted from the efflux values obtained with the cholesterol acceptors.

### Total cholesterol quantification

Cells were seeded in 6 well plates and treated with saline or 50 nM HDL NPs for 24 hours. Following treatment, cells were lysed and the total cholesterol content quantified using the Amplex Red Cholesterol Assay (ThermoFisher) and a BioTek Synergy 2 microplate reader. Protein content was measured using the BCA assay. Data are presented as μg cholesterol per mg protein.

### WST-1 viability assay

Cells were seeded in 96-well plates at a density of 3,000 cells per well. Cells were seeded in medium containing HDL NPs, hHDL (Millipore), or vehicle control (PBS) at the indicated doses. After incubation for 5 days at 37 °C in 5% CO_2_, the WST-1 reagent (Roche) was added and plates were analysed according to the manufacturer’s instructions using the Synergy HT plate reader with Gen5 software (BioTek).

### Soft agar assay

Cells were seeded in soft agar at a density of 2,500 cells per well. Medium containing HDL NPs or vehicle control (PBS) was added at the indicated doses. Cells were incubated for 7 days at 37 °C in 5% CO_2_, colony formation was assessed using the CyQUANT GR Dye (Cell Biolabs) and plates were analysed according to the manufacturer’s instructions using the Synergy HT plate reader with Gen5 software (BioTek).

### Sphere forming assay and ELDA

3-D CSC cultures were dissociated into single cells and seeded at the indicated cell densities into round-bottom 96-well plates (Greiner Bio-One) in CSC medium containing HDL NPs or vehicle control (PBS) at the indicated doses. Spheres were incubated for 7 days at 37 °C in 5% CO_2_ followed by staining with acridine orange as previously described^36^. Sphere size was assessed using a Cytation 3 Cell Imaging Multi-Mode Reader. Spheres ≥100 μm diameter were scored positively for sphere formation. CSC frequency was determined using extreme limiting dilution analysis (ELDA)^37^.

### Statistics

All statistics were performed using GraphPad Prism 6.0 for Mac. The student’s t-test and 1-way ANOVA tests were used where appropriate.

### Data availability

The datasets analysed during this study are available from the GlioVis portal (https://gliovis.shinyapps.io/GlioVis/) and Broad-Novartis Cancer Cell Line Encyclopedia (CCLE) (http://www.broadinstitute.org/ccle).

## Electronic supplementary material

Supplemental Figures
