# Supplementary material for: HDL nanoparticles targeting sonic hedgehog subtype medulloblastoma
Source: Sci Rep. 2018 Jan 19;8:1211. doi: 10.1038/s41598-017-18100-8 (PMC5775338; doi:10.1038/s41598-017-18100-8)
Supplement: Supplementary file 1 — Supplemental Figures [file 41598_2017_18100_MOESM1_ESM.doc]

**HDL nanoparticles targeting sonic hedgehog subtype medulloblastoma**

Jonathan B. Bell, Jonathan S. Rink, Frank Eckerdt, Jessica Clymer, Stewart Goldman, C. Shad Thaxton, and Leonidas C. Platanias

**Supplemental Figure 1-3**

**Figure S1. Inverse correlations between *SCARB1* and the non-SHH genes *RPIG1*, *EOMES*, and *LEMD1*. (a-c)** Gene expression data were analysed by correlation analysis. *SCARB1* is compared with the three genes found to be most underexpressed in *SCARB1*-low medulloblastoma samples: *RPGIP1* (a), *EOMES* (b), and *LEMD1* (c). Pearson’s correlation coefficients are shown, ****P ≤ 0.0001.


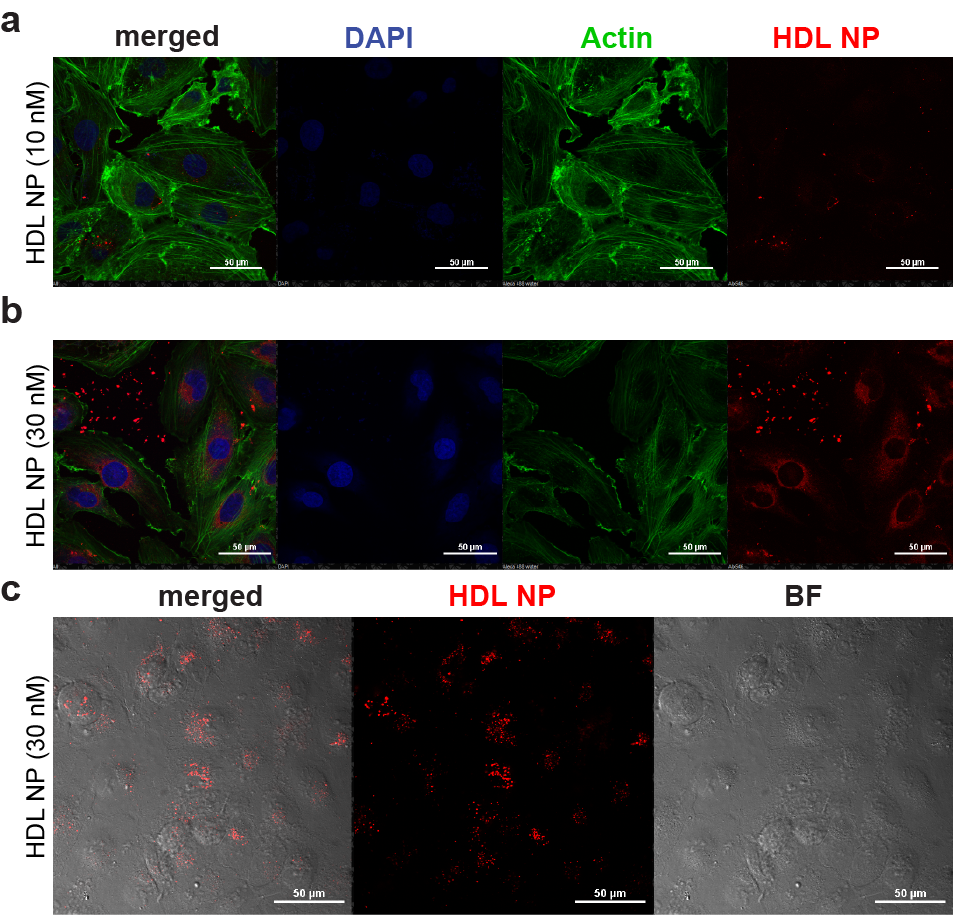


**Figure S2. Dose-dependent uptake of HDL NPs in medulloblastoma cells. (a-b)** Imaging by confocal laserscan microscopy of DAOY cells treated with 10 nM or 30 nM DiI-labeled HDL NPs for 24 hours. Images show cell nuclei (blue), actin (green) and HDL NPs (red). **(c)** Live cell imaging by confocal laserscan microscopy of DAOY cells treated with 30 nM DiI-labeled HDL NPs for 24 hours (red) with bright field (BF) are shown. Scale bar = 50 μM.

**Figure S3. Effect of HDL NPs on viability in non-SHH subtype D556 medulloblastoma cells.** D556 cells were treated with increasing concentrations of HDL NPs for 5 days followed by analysis with the WST-1 cell proliferation assay. Data represents means ± SEM of 3 independent experiments.
